# Protein-sol pKa: prediction of electrostatic frustration, with application to coronaviruses

**DOI:** 10.1093/bioinformatics/btaa646

**Published:** 2020-07-19

**Authors:** Max Hebditch, Jim Warwicker

**Affiliations:** School of Biological Sciences, Faculty of Biology, Medicine and Health, Manchester Institute of Biotechnology, Manchester M1 7DN, UK; School of Biological Sciences, Faculty of Biology, Medicine and Health, Manchester Institute of Biotechnology, Manchester M1 7DN, UK

## Abstract

**Motivation:**

Evolution couples differences in ambient pH to biological function through protonatable groups, in particular, those that switch from buried to exposed and alter protonation state in doing so. We present a tool focusing on structure-based discovery and display of these groups.

**Results:**

Since prediction of buried group pKas is computationally intensive, solvent accessibility of ionizable groups is displayed, from which the user can iteratively select pKa calculation centers. Results are color-coded, with emphasis on buried groups. Utility is demonstrated with benchmarking against known pH sensing sites in influenza virus hemagglutinin and in variants of murine hepatitis virus, a coronavirus. A pair of histidine residues, which are conserved in coronavirus spike proteins, are predicted to be electrostatically frustrated at acidic pH in both pre- and post-fusion conformations. We suggest that an intermediate expanded conformation at endosomal pH could relax the frustration, allowing histidine protonation and facilitating conformational conversion of coronavirus spike protein.

**Availability and implementation:**

This tool is available at http://www.protein-sol.manchester.ac.uk/pka/.

## 1 Introduction

Since pKas underlie pH-dependent phenomena in biology, their prediction has received significant attention, largely through continuum electrostatics methods (Alexov *et al.*, 2011). We have contributed a server for predicting pH and ionic strength dependence with a Debye–Hückel (DH) model that accounts for solvent exposed groups, which are generally in the great majority (Hebditch and Warwicker, 2019). However, conformational change often depends on the electrostatic frustration (destabilization) that develops when a buried group cannot ionize at a pH where it would in a more solvent accessible conformation (Narayan and Naganathan, 2018). We reasoned that a web tool focusing on buried ionizable groups would be useful for studying pH-dependent conformational change, and have adapted our existing mixed finite difference Poisson–Boltzmann (FDPB) and DH model. The FDPB part of this implementation uses a previously defined separation of contributions from continuum electrostatics (Bashford and Karplus, 1990) and incorporates Monte Carlo sampling of protonation states (Beroza *et al.*, 1991). Our aim is to produce a tool that allows rapid screening of structures by non-expert users, which is therefore complementary to the more detailed and computationally intensive investigation, for example, with constant pH molecular dynamics (Chen *et al.*, 2014), available to expert users. After benchmarking against measured data, the server is demonstrated with coronaviruses, some of which use the endocytotic pathway for membrane fusion, whereas others fuse at the plasma membrane. We focus on the pre- to post-fusion conformational changes in the S2 part of the spike protein, that mediates membrane fusion (Heald-Sargent and Gallagher, 2012).

## 2 Materials and methods

We have sought to limit FDPB/DH run time for pKa predictions (Warwicker, 2009) to about two minutes processing. Upon upload of a structure, the user is presented with a color-coded display (Rose *et al.*, 2018) of solvent accessible surface area (ASA) values for ionizable groups. A user iteratively specifies centers, around which pKa calculations are made for spheres of radius 25 Å (about the size of lysozyme). Edge effects do not have a big effect on predicted pKas toward the sphere center. Results for Asp, Glu, Lys, Arg and His accumulate as more centers are added, and are color-coded to show whether a group is stabilizing or destabilizing, assessed from the difference between calculated and intrinsic pKa (capped at -5 and 5). The ΔpKas observed at a single site are representative of interactions within a charge network. In a simple case, for just two interacting groups, each of the two ΔpKas measures the entire contribution of that pair interaction to stability. Users may either use ionizable group ASA/burial or literature knowledge of interesting sites, to select pKa calculation centers. It is envisaged that the server will allow a user to quickly survey a set of structures for potential pH-dependence hotspots, rather than provide a great depth of analysis for each structure.

## 3 Results

### 3.1 Comparison with experimental data

**Fig. 1.**
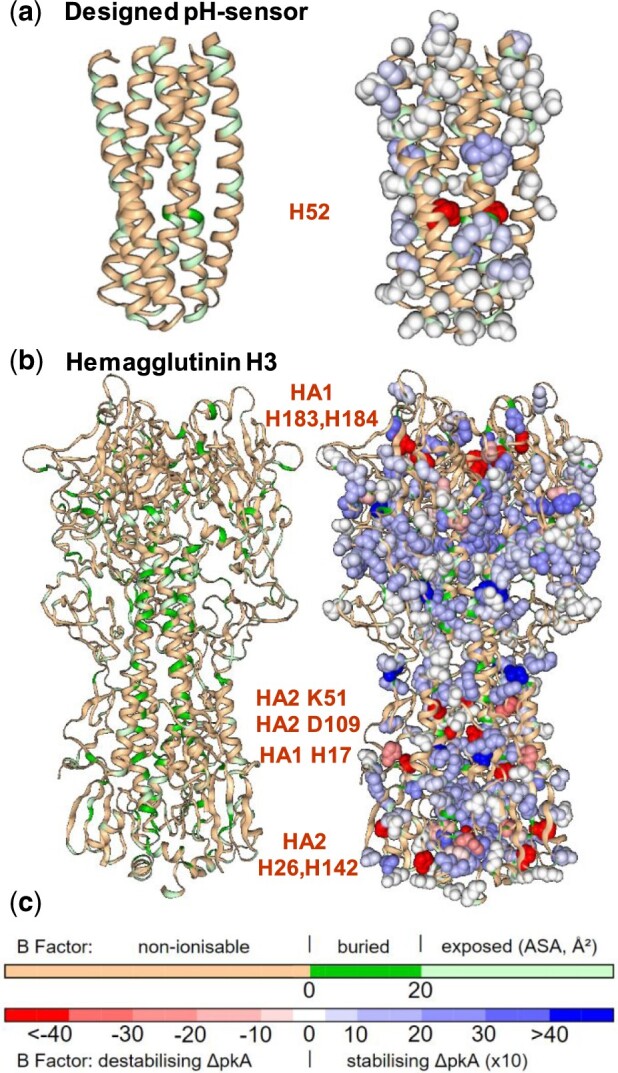
Burial and pKas. (**a**) In a designed pH sensor (6msr), H52 is both the sole substantially buried (dark green, left) and destabilized (red, right) ionizable group. (**b**) For hemagglutinin (2viu), many buried ionizable groups (left) do not map to electrostatic frustration (right, listed). (**c**) Color scales for burial and ΔpKas

To assess the mapping of burial to electrostatic frustration, two quite different trimer examples are discussed (Fig. 1). For one of a set of designed pH sensors, pRO-2.5 (6msr, Boyken *et al.*, 2019), the single (trimeric) signal for electrostatic frustration (H52) identifies with the only substantially buried region (dark green, Fig. 1a, c). H52 is also the designed pH-sensing residue (Boyken *et al.* 2019). Elsewhere on the surface, ionizable groups range from minimally to significantly stabilizing (more blue) ΔpKas, part of the balance between folded state interactions and acid pH destabilization noted in the design study (Boyken *et al.*, 2019). A similar balance has been noted (Daniels, 1985) for the larger and more complex hemagglutinin trimer, HA1-HA2 cleaved H3 (2viu, Fleury *et al.*, 1998). Here, substantial burial does not necessarily lead to electrostatic frustration (Fig. 1b), emphasizing the role of pKa calculations in discriminating the balance of interactions, in particular, destabilizing desolvation versus stabilizing charge networks. Most of the identified groups are known in the extensive literature for this H3 hemagglutinin. Of the adjacent H183, H184 grouping in HA1, H184 has been found to influence the fusion pH, tested in H5 (Mair *et al.*, 2014), whilst H183 is critical for receptor binding (Chen *et al.*, 2018). HA2 K51 is reported to abrogate sensitivity to a conformational change inhibitor, when mutated to arginine in the H1 sub-type (Yoshimoto *et al.*, 2000). HA2 D109 is buried and adjacent to the fusion peptide, upon cleavage of HA0 to HA1-HA2 (Trost *et al.*, 2019). Also close to the fusion peptide, mutation of HA1 H17 alters the fusion pH (Thoennes *et al.*, 2008). H142 of HA2 has been suggested to play a role in pH-dependent fusion (Kampmann *et al.*, 2006). Although HA2 H26 is the single residue from our calculation of electrostatic frustration that is not mentioned in the literature, it is highly conserved (Lee *et al.*, 2018). In summary, comparison with measurement, for one of the most studied pH-dependent systems, demonstrates that burial, although generally necessary, is insufficient to predict electrostatic frustration. Further, the electrostatically frustrated groups that are identified by our model compare well with known sites.

### 3.2 Application to coronaviruses

**Fig. 2.**
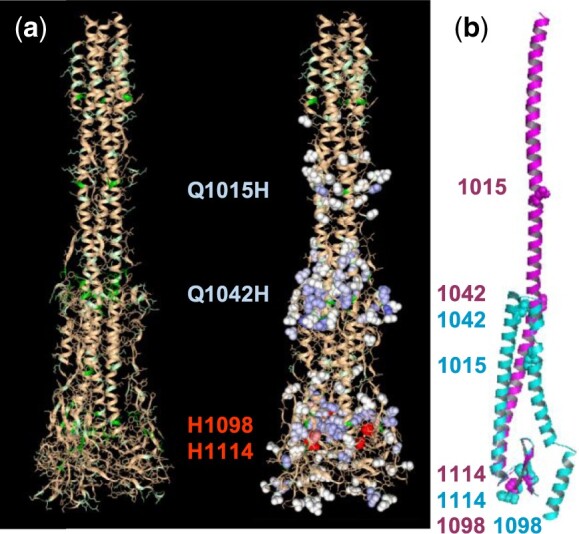
Coronavirus in the pKa web server. (**a**) The post-fusion structure of MHV A59 (6b3o) with ionizable group burial (left) and pKa calculations around selected centers (right, color codes Fig. 1c). (**b**) Segments of pre- (3jcl, cyan) and post-fusion (6b3o, magenta) MHV A59 structures. Residues of interest are indicated throughout

Although there are about 40 structures of pre-fusion coronavirus spike proteins, there is just one post-fusion structure available (April 2020) that extends beyond the helical fusion core, for murine hepatitis virus (MHV) strain A59 (6b3o, Walls *et al.*, 2017). A pre-fusion structure for MHV A59 is also available (3jcl, Walls *et al.*, 2016). In a variant of mouse hepatitis virus type 4 (MHV4), spike protein Q1015H, Q1042H (MHV A59 numbering) and one further mutation (L to R) render the virus pH-dependent, via the endocytotic pathway (Gallagher *et al.*, 1991). Modeling the mutations to histidine in the MHV A59 background, we find they are predicted to be buried in the pre-fusion form (destabilizing, not shown) and exposed in the post-fusion structure (not destabilizing, Fig. 2a). The two panels in Figure 2a demonstrate sequential use of the web tool, first (left) displaying burial (by color-coding given in Fig. 1c), and second (right) showing ΔpKa calculations around selected centers. Selected residue numbers in Figure 2a are also color-coded by the presence (red) or absence (blue) of electrostatic frustration in the post-fusion spike protein. Our results are consistent with the relief of electrostatic frustration at endosomal pH biasing conformation away from the pre-fusion structure. The story is a little more complicated, since the MHV4 variant also loses fusion activity at neutral pH (Gallagher *et al.*, 1991), which could be due to additional stabilization of the pre-fusion form with pH-independent histidine interactions.

Figure 2b shows equivalent parts (972–1118) of a monomer from pre- and post-fusion MHV A59, structurally aligned through a small common core around 1098/1114. Extending from the structurally aligned segments are helices that demonstrate the extensive spike protein structural changes that go along with cell fusion. Whereas the helical region wraps back around the aligned core in the pre-fusion structure, it extends upwards post-fusion, carrying the fusion peptide toward its target membrane, shown by the relative locations of 1015. Switching to conserved histidines and general coronavirus features, only two are present across the spike proteins of coronaviruses, H1098 and H1114 (MHV A59 numbering). In 37 of 38 pre-fusion coronavirus spike protein structures, as well as the post-fusion structure, H1098 and H1114 are buried and predicted to be destabilizing upon exposure to acidic pH (Fig. 2a, red spacefill). If these conserved histidines are electrostatically frustrated in both pre- and post-fusion conformations at endosomal pH, they would not bias toward either form. However, to allow the extensive changes exemplified in Figure 2b, it is possible that the core region around H1098/H1114 loosens. If H1098 and/or H1114 were solvent exposed and protonated, then relief from frustration in a conformational intermediate could play a role in facilitating transfer between post- and pre-fusion structures in coronaviruses that use the endocytotic pathway, including SARS-CoV-2 (Ou *et al.*, 2020). In this proposal, H1098/H1114 assistance in crossing the pre- to post-fusion conformational barrier would be available to those viruses that are unable to fuse at the plasma membrane. Interestingly, both H1098A and H1114A mutations in MHV A59 prevented virus growth (Li *et al.*, 2018), perhaps indicative of (pH-independent) packing stabilizations in their buried environments, so that evolutionary retention for fusion could be afforded by a more direct structural imperative. This would be in line with the coupling of factors that determine infection pathways, including spike protein stability, receptor binding, proteolytic cleavage, as well as endosomal pH (Heald-Sargent and Gallagher, 2012; Millet and Whittaker, 2015). If the proposed loosening around the conserved post- and pre-fusion cores is borne out, then although it may not be universally necessary (in pH-dependent entry), it could be the basis for a novel, albeit transient, coronavirus drug target.

Our web tool will allow users to look for ionizable groups that could mediate pH-dependence in coronaviruses and other systems.
